# Carers’ Attitudes Towards Prescribing of Psychotropic Medications in Children and Adolescents With Intellectual Disabilities in Malta

**DOI:** 10.1192/bjo.2025.10117

**Published:** 2025-06-20

**Authors:** Noemi Cortis, Sian Edney, Rosemarie Sacco

**Affiliations:** ^1^Cardiff University, Cardiff, United Kingdom; ^2^Mental Health Services Malta, Malta

## Abstract

**Aims:** This study’s primary aim was to explore carers’ views of psychotropic medication prescribing in children and adolescents with intellectual disability (ID) followed up at Malta’s Intellectual Disability Clinic (IDC). It also aimed to identify carers’ views on nonpharmacological management of challenging behaviour (CB), experiences of Malta’s Child IDC and perceptions of mental illness in children with ID.

**Methods:** A literature review was carried out to gather data on previous research on the topic. Ethical and Departmental approval were obtained. Purposive sampling was used to recruit thirteen caregivers of children and adolescents with a diagnosis of a mild, moderate, or severe ID and/or moderate or severe autism spectrum disorder who were prescribed psychotropic medication and attending the Child IDC. Semi-structured interviews were carried out using an interview guide written by the primary researcher. These were recorded digitally and then transcribed verbatim. A reflexive and inductive thematic analysis approach was taken to analyse and interpret data.

**Results:** Seven themes were generated, with the predominant theme being ‘Medication is a necessary evil’. The other themes were 1) Medication helps them cope, 2) A last resort, 3) The doctor knows best, 4) Medication falls short of expectations, 5) A cure for all the family, 6) Medication: The missing piece to society’s puzzle.

Overall, psychotropic medication was perceived as beneficial, as it improved the children’s quality of life, their functioning within their roles within the family and within society. Parents experienced a lot of barriers when accessing services, social benefits and struggled with inclusion in society. Parents were aware that medication did not come without adverse effects, some of which proved to be of detriment to these children and their families.

**Conclusion:** This study highlighted the need for increased psychoeducation and parental support. Parental burnout, child’s distress, and fear of exclusion from society may contribute to perceived need for medication. The lack of streamlined services and familial support further exacerbate parental struggles, leading them to resort to medication. Further research is needed to discover prevalence rates of mental health disorders in the Maltese cohort. Improvements to the IDC include provision of family counselling, parenting programmes and support groups. Access to Positive Behaviour Support at the Child IDC Services may allow for alternatives prior to resorting to psychotropic medication for CB.

